# Laser-Induced Changes in Root Dentin Microhardness: Evidence From a Systematic Review

**DOI:** 10.7759/cureus.106888

**Published:** 2026-04-12

**Authors:** Ansha Jasmin S, Seema Merwade, Kiran Kumar Neelakantappa, Andria Dsouza, Vaishnavi Kashim

**Affiliations:** 1 Conservative Dentistry and Endodontics, Government Dental College and Research Institute, Bangalore, IND

**Keywords:** dentin, endodontics, laser irradiation, microhardness, smear layer

## Abstract

Dentin plays a critical role in tooth strength and function, yet conventional rotary instrumentation produces a smear layer that impedes bonding and cleaning. Laser technology has been introduced as an alternative for dentin conditioning, offering precise ablation and minimal thermal damage when properly applied. However, variations in laser type and parameters have led to inconsistent findings regarding their effects on dentin. This systematic review aimed to evaluate the effects of laser irradiation on dentin microhardness, smear layer removal, and surface morphology in in-vitro studies, focusing on Er:YAG, Er,Cr:YSGG, Nd:YAG and diode laser systems. A comprehensive search of PubMed, Scopus, Embase, Web of Science, and Google Scholar was conducted up to June 15, 2025. Eligible in vitro studies investigated dentin’s structural, compositional, or mechanical changes following laser irradiation. Data were extracted on laser parameters, outcomes, and bias using the Joanna Briggs Institute checklist. Due to heterogeneity, findings were narratively synthesized. Thirteen studies published between 2012 and 2025 met the inclusion criteria. Diode and Er,Cr:YSGG lasers showed superior smear layer removal and enhanced surface cleanliness. Er:YAG lasers demonstrated effective cleaning with minimal damage. Nd:YAG and high-powered Er,Cr:YSGG reduced microhardness and caused morphological changes. Most studies had low to moderate risk of bias. Diode and erbium-based lasers effectively enhance smear layer removal and preserve dentin integrity when used under controlled parameters. Optimized laser protocols show strong potential as adjunctive tools for safer and more efficient dentin conditioning in restorative and endodontic practice.

## Introduction and background

Dental hard tissues, particularly enamel and dentin, are vital for the structural and functional integrity of teeth [1]. Dentin is a hydrated biological composite composed mainly of mineral, collagen, and water. It provides resilience, supports enamel, and protects the underlying pulp [2]. Preserving its structural and mechanical integrity is essential for the long-term success of restorative procedures [3].

Conventional cavity preparation using rotary instruments is effective but has several drawbacks [4]. It can produce vibration, heat, and discomfort for patients. More importantly, it creates a smear layer that blocks dentinal tubules and may interfere with adhesive bonding [5]. To overcome these limitations, laser technology has been introduced as an alternative approach for cavity preparation, dentin conditioning, and surface modification.

Lasers offer distinct advantages, including precise tissue removal, reduced pain perception, and minimal thermal side effects when used under proper parameters. Among the different types, erbium lasers (Er:YAG and Er,Cr:YSGG) are most commonly used for hard tissue applications because of their strong affinity for water and hydroxyapatite [6]. They are capable of efficient ablation of enamel and dentin with limited collateral damage. Diode lasers, conversely, are more widely used in soft-tissue and endodontic applications, but they are increasingly being studied for their potential effects on dentin when used at controlled energy levels [7].

The interaction between laser energy and dentin can lead to both beneficial and undesirable changes. Depending on the energy density, pulse duration, and cooling, laser irradiation can modify surface morphology, remove or reduce the smear layer, and alter the microhardness and mineral composition of dentin [8]. Some studies report that appropriate laser settings improve dentin bonding and cleanliness by opening tubules and creating micro-retentive surfaces [9-11]. Others, however, have observed structural damage such as collagen denaturation, cracks, or mineral loss when excessive energy is used [12,13].

Over the past two decades, numerous in vitro studies have investigated the effects of different laser types and parameters on dentin morphology and properties. Despite this growing body of evidence, the findings remain inconsistent and sometimes contradictory. Differences in study design, laser systems, and assessment methods make it difficult to compare results and reach a consensus on safe and effective parameters [14-26]. A systematic synthesis of existing data is therefore needed to clarify how laser irradiation influences dentin’s structure and mechanical behavior, and to guide clinical use in restorative and endodontic procedures.

The objective of this systematic review is to evaluate the effects of laser irradiation on the structure, composition, and mechanical properties of dentin as reported in in vitro studies. Specifically, the review aims to determine how different laser types, such as Er:YAG, Er,Cr:YSGG, Nd:YAG and diode lasers, and their operating parameters affect dentin microhardness, surface morphology, and mineral composition.

## Review

Methodology

Protocol and Reporting Guidelines

The PRISMA (Preferred Reporting Items for Systematic Reviews and Meta-Analysis) standards were followed in the design and execution of this systematic review. Under the ID CRD420251136026, the review methodology was prospectively filed with the International Prospective Register of Systematic Reviews (PROSPERO). Prior to guaranteeing rigor and openness, all methodological choices, including search, selection, and synthesis tactics, were established.

Eligibility Criteria

This review included in vitro experimental studies that evaluated the effects of laser irradiation on human dentin and in vitro animal studies with relevant findings. Studies were eligible if they investigated changes in dentin structure, composition, or mechanical properties (e.g., microhardness, roughness, mineral content, or morphology) following exposure to dental lasers. Both Erbium lasers (Er:YAG, Er,Cr:YSGG), Nd:YAG, and diode lasers were included.

Studies were excluded if they were reviews, case reports, conference abstracts, in vivo animal studies, or did not assess dentin-related outcomes. Studies that investigated only soft-tissue effects, caries detection, or pulp vitality without dentin analysis were also excluded.

For synthesis, studies were grouped according to laser type (Er:YAG, Er,Cr:YSGG, Nd:YAG or Diode) and primary outcome (microhardness, compositional changes, or morphological alterations).

Information Sources

A comprehensive electronic search was conducted in PubMed, Scopus, Embase, Web of Science, and Google Scholar databases. Additional sources included ClinicalTrials.gov, ProQuest Dissertations, and reference lists of eligible studies to identify any missed publications.

The final database search was completed on June 15, 2025, with no language or date restrictions. Manual searches were also performed on the websites of the Journal of Conservative Dentistry, Lasers in Medical Science, and Photomedicine and Laser Surgery for relevant in-vitro studies not indexed in databases.

Search Strategy

Search terms were developed using Medical Subject Headings (MeSH) and free-text keywords related to dentin and laser irradiation. The PubMed search string was:

("laser irradiation" OR "Er:YAG" OR "Er,Cr:YSGG" OR "Nd:YAG" OR "diode laser") AND ("dentin" OR "dentine") AND ("microhardness" OR "morphology" OR "composition" OR "smear layer" OR "calcium" OR "surface roughness")

Equivalent search strings were adapted for other databases. Boolean operators, truncations, and filters were adjusted according to each database interface. Reference lists of all included articles were screened manually for additional eligible studies.

Selection Process

Two independent reviewers screened all titles and abstracts retrieved from the search. Full texts were then evaluated to confirm eligibility. Discrepancies were resolved through discussion and consensus, with a third reviewer consulted when necessary.

Data Extraction and Outcomes Assessed

Data were extracted independently by two reviewers using a standardized data extraction form developed for this review. Extracted items included study characteristics (author, year, country), laser type and parameters, sample size, dentin source, outcomes assessed, and main results.

Where information was incomplete or unclear, the reviewers attempted to interpret missing data based on the methods and figures described in the articles. No direct contact with authors was required. Disagreements in data extraction were resolved by consensus.

The primary outcomes were dentin microhardness, mineral composition (Ca, P ratio), surface morphology, and collagen or structural changes following laser exposure. All quantitative and qualitative results compatible with these domains were recorded, regardless of measurement technique or time point.

Secondary variables included laser wavelength, pulse duration, power or energy density, irradiation mode, and cooling system. Assumptions about unclear data were noted, and missing quantitative details were treated descriptively.

Risk of Bias Assessment

Risk of bias for included in vitro studies was evaluated using the Joanna Briggs Institute (JBI) Critical Appraisal Checklist for Experimental Studies, which is suitable for laboratory-based research. Each study was assessed independently by two reviewers across domains including randomization, laser parameter standardization, outcome measurement reliability, blinding, statistical analysis, and completeness of data.

Disagreements were resolved by consensus. No automation or software-assisted tools were used. Studies were categorized as low, moderate, or high risk of bias based on the overall assessment.

Effect Measures

For quantitative outcomes such as microhardness or mineral content, the effect measure used was mean difference (Δ) between laser-irradiated and control samples. When applicable, standard deviations and p-values were extracted to describe the direction and magnitude of change. For qualitative outcomes such as morphological changes under scanning electron microscopy (SEM), results were presented descriptively.

Synthesis Methods

Studies were tabulated according to laser type and outcome to facilitate comparison. Descriptive synthesis was performed because of the heterogeneity in study designs, laser parameters, and measurement techniques.

No formal meta-analysis was conducted. Quantitative results were summarized as mean changes or relative comparisons when units and methods were consistent. Visual presentation was achieved through summary tables and comparative charts of laser parameters versus dentin outcomes.

Where heterogeneity was evident, findings were narratively synthesized, emphasizing consistencies and contradictions between studies. Subgroup or sensitivity analyses were not performed due to variability in reporting formats and insufficient comparable data.

Results

Study Selection

**Figure 1:**
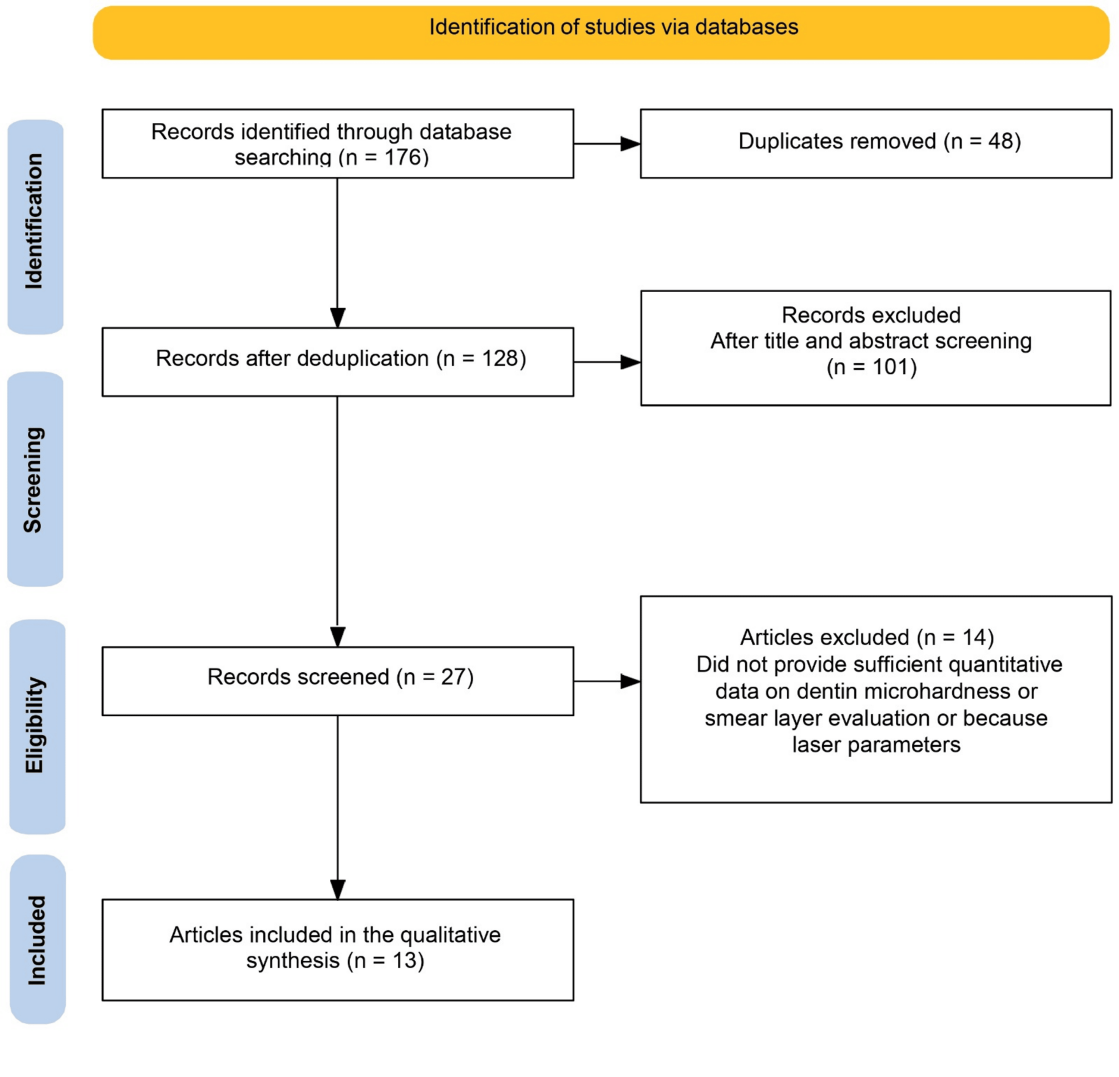
PRISMA Workflow for Selection of Studies Included PRISMA: Preferred Reporting Items for Systematic Reviews and Meta-Analysis

The database search initially yielded 176 records from PubMed, Scopus, Web of Science, and Google Scholar. After removing 48 duplicates, 128 titles and abstracts were screened. A total of 101 studies were excluded at this stage for not meeting the inclusion criteria, primarily due to evaluating non-laser techniques, non-dentin substrates, or lacking comparative design. Subsequently, 27 full-text articles were assessed for eligibility. Of these, 14 studies were excluded because they did not provide sufficient quantitative data on dentin microhardness or smear layer evaluation, or because laser parameters were inadequately described. Ultimately, 13 studies met all inclusion criteria and were included in the qualitative synthesis. The study selection process is summarized in a PRISMA-style flow diagram, illustrating identification, screening, eligibility, and inclusion stages (Figure 1).

Study Characteristics

Thirteen in vitro studies published between 2012 and 2025 were included. The studies examined a variety of laser systems, including diode (808-980 nm), Nd:YAG (1064 nm), Er:YAG (PIPS and SWEEPS), and Er,Cr:YSGG (2780 nm). Sample sizes ranged from 18 to 80 specimens, and tooth types included in vitro human, bovine and canine dentin samples. Most studies were conducted under controlled laboratory conditions comparing laser-irradiated samples with conventional irrigation protocols such as sodium hypochlorite (NaOCl), ethylenediaminetetraacetic acid (EDTA), MTAD, or non-irradiated controls. The primary outcomes measured were microhardness (Knoop or Vickers), smear layer removal, surface morphology assessed by SEM or energy-dispersive X-ray spectroscopy (EDS), and fracture resistance. Among these, nine studies evaluated microhardness, five studies assessed smear layer and surface characteristics, and one study (Elmiligy et al., [17]) evaluated fracture resistance. The majority of studies used diode lasers (7/13), followed by Er,Cr:YSGG (3/13), Nd:YAG (2/13), and Er:YAG (1/13).

Results of Individual Studies

The findings varied according to the laser type and application parameters. Microhardness outcomes were inconsistent across studies. Some studies reported increased microhardness following diode laser irradiation, whereas others demonstrated reductions in dentin hardness after Nd:YAG and Er,Cr:YSGG exposure. Elmiligy et al. [17] demonstrated increased fracture resistance without microhardness alteration using a diode laser.

Smear layer removal was consistently improved in studies using diode and Er,Cr:YSGG lasers observed minimal surface improvement. SEM and EDS analyses revealed that diode irradiation produced amorphous surfaces, Er,Cr:YSGG created crater-like patterns, and Nd:YAG caused surface fusion, reflecting energy-dependent morphological alterations. Braun et al. [20] found that continuous diode laser application caused more dentinal microcracks than interval mode, indicating that intermittent irradiation minimizes thermal damage.

Recent studies demonstrated that combining laser application with fluoride treatment improved dentin acid resistance and limited hardness loss following demineralization cycles. Table 1 presents a comprehensive summary of the included studies.

**Table 1:** Summary of Included Studies

No.	Author (Year)	Laser Type/Parameters	Sample & Design	Comparator	Outcomes Measured	Main Findings
1	Saraswathi et al. (2012) [14]	Diode 940 nm	30 premolars, in-vitro	Conventional irrigants	Smear layer, Ca/P ratio	Improved smear layer removal with diode laser; Ca/P unchanged
2	Viapiana et al. (2012) [15]	Diode 980 nm, 1.5 W & 3.0 W, 20 s helicoidal	72 canines, three irrigants	Non-irradiated dentin	Knoop microhardness (30–300 µm)	Laser groups showed higher microhardness than controls; no difference between powers
3	Al-Omari & Palamara (2013) [16]	Nd:YAG (2 W) & Er,Cr:YSGG (3.5/4.5 W)	30 molar dentin discs (split halves)	Non-irradiated halves	Vickers microhardness	Both lasers reduced microhardness; Er,Cr:YSGG 3.5 W > 4.5 W > Nd:YAG
4	Elmiligy et al. (2012) [17]	Diode 980 nm, 2 W	60 premolars, in-vitro	No laser	Fracture resistance, microhardness	Laser increased fracture resistance; no microhardness change
5	de Macedo et al. (2015) [18]	Nd:YAG 1064 nm 1.5 W; Diode 980 nm 2 W	25 bovine roots, 5 groups	EDTA & manual agitation	Knoop hardness + surface roughness	Both lasers decreased hardness; highest roughness in Nd:YAG > diode
6	Alhadi et al. (2019) [21]	Diode 810 nm, continuous	18 premolars, in-vitro	NaOCl + EDTA	Smear layer (SEM), EDX composition	Slight mineral loss; no major smear layer improvement
7	Lopes et al. (2018) [19]	Diode 980 nm; Nd:YAG 1064 nm; Er,Cr:YSGG 2780 nm (1.5 W 20 s)	25 canines, 5 groups	Non-laser irrigation	EDS + SEM	No chemical changes (EDS); Diode = amorphous surface, Er,Cr:YSGG = craters, Nd:YAG = fusion
8	Braun et al. (2018) [20]	Diode 970 nm, constant vs interval mode (1.5 W, 15 Hz)	40 human roots, 4 groups	Ca(OH)₂ & control	Microcrack formation (optical microscopy)	Constant mode → most cracks; interval mode ≈ control → safest
9	Quteifani (2022) [22]	Diode 980 nm	36 premolars, in-vitro	MTAD (non-laser)	Microhardness	MTAD + laser = less hardness loss vs MTAD alone
10	Aboudoura et al. (2024) [23]	Er,Cr:YSGG (Waterlase MD)	40 premolars	NaOCl + EDTA	Smear layer, microhardness	Laser decreased hardness; combo with AgNP similar to control
11	Patil et al. (2025) [26]	Diode 808 nm, 1 W, 30 s	30 extracted premolars (5 groups)	NaOCl, EDTA, saline controls	Vickers microhardness	Laser + EDTA showed highest microhardness reduction; diode irradiation decreased hardness vs controls
12	Mobarak et al. (2025) [24]	Er:YAG (PIPS, SWEEPS)	44 premolars	Ultrasonic (PUI), conventional (CI)	Microhardness, dye penetration	SWEEPS preserved microhardness; both lasers ↑ penetration depth
13	de Menezes et al. (2025) [25]	Er,Cr:YSGG 0.25 W 5 Hz ± F⁻; Diode 980 nm 2 W 2 Hz ± F⁻	80 bovine incisors, 8 groups + pH cycling	No-treatment & fluoride	Knoop hardness (pre/post cariogenic challenge)	Laser ± fluoride limited hardness loss; both improved acid resistance

Synthesis of Results

Given the methodological diversity among studies, a narrative synthesis was performed. Diode and Er:YAG lasers generally maintained or enhanced dentin microhardness, while Nd:YAG and Er,Cr:YSGG tended to reduce it at higher energy levels. In terms of smear layer removal, diode and Er,Cr:YSGG lasers provided superior cleaning efficacy compared to conventional irrigants. One study suggested that diode lasers can enhance fracture resistance, though this finding requires further confirmation. The direction of effects across studies was consistent, showing beneficial outcomes for smear layer removal and acceptable effects on microhardness when appropriate parameters were applied.

Risk of Bias in Included Studies

The overall risk of bias across the 13 studies ranged from low to moderate, with one study rated as high risk (de Macedo et al., [18]). Domains evaluated included randomization, standardization of laser parameters, outcome reliability, blinding, statistical analysis, completeness of data, and overall bias. Six studies (Viapiana et al., [15]; Elmiligy et al., [17]; Lopes et al., [19]; Braun et al., [20]; Aboudoura et al., [23]; Mobarak et al., [24]) demonstrated a low overall risk of bias. Five studies were judged to have a moderate risk, primarily due to limited randomization or lack of blinding, and one had high risk due to methodological weaknesses. Statistical analyses were appropriate in all studies, and no data incompleteness was reported. Table 2 summarizes risk of bias assessment of included studies.

**Table 2:** Risk of Bias Assessment using the JBI Checklist JBI: Joanna Briggs Institute

No.	Study (Year)	Randomization of Samples	Laser Parameter Standardization	Outcome Measurement Reliability	Blinding of Assessors	Statistical Analysis	Completeness of Data	Overall Risk of Bias
1	Saraswathi et al. (2012) [14]	Low	Moderate	Low	Moderate	Low	Low	Moderate–Low
2	Viapiana et al. (2012) [15]	Low	Low	Moderate	Moderate	Low	Low	Low
3	Al-Omari & Palamara (2013) [16]	Moderate	Low	Moderate	Moderate	Low	Low	Moderate
4	Elmiligy et al. (2012) [17]	Low	Low	Low	Moderate	Low	Low	Low
5	de Macedo et al. (2015) [18]	High	Low	Moderate	High	Low	Low	High
6	Lopes et al. (2018) [19]	Low	Low	Moderate	Moderate	Low	Low	Low
7	Braun et al. (2018) [20]	Low	Low	Moderate	Moderate	Low	Low	Low
8	Alhadi et al. (2019) [21]	Moderate	Low	Low	High	Low	Low	Moderate
9	Quteifani (2022) [22]	Moderate	Low	Low	Moderate	Low	Low	Low–Moderate
10	Aboudoura et al. (2024) [23]	Low	Low	Low	Moderate	Low	Low	Low
11	Mobarak et al. (2025) [24]	Low	Low	Low	Low	Low	Low	Low
12	de Menezes et al. (2025) [25]	Moderate	Low	Moderate	Moderate	Low	Low	Moderate
13	Patil et al. (2025) [26]	Low	Low	Moderate	Moderate	Low	Low	Moderate

Discussion

The results of this systematic review provide important insights into the potential role of laser-assisted endodontic techniques in modifying dentin surface characteristics, improving smear layer removal, and maintaining or enhancing microhardness. Across the thirteen included studies, a consistent pattern emerged: lasers, particularly diode, Er:YAG, and Er,Cr:YSGG systems, demonstrated beneficial effects when used under controlled conditions. These findings are broadly consistent with previous research emphasizing the adjunctive advantages of laser activation in root canal treatment and confirm that the specific parameters of laser application such as wavelength, energy, pulse mode, and exposure duration play a decisive role in determining clinical outcomes [5,6]. In the context of existing evidence, the results of this review reinforce the growing understanding that lasers can optimize dentin conditioning, improve irrigant penetration, and enhance the overall effectiveness of root canal disinfection [7,8].

Effect of Different Laser Systems

Diode lasers: When placed in the context of earlier reviews and experimental work, the results affirm that diode lasers operating between 808 and 980 nm can effectively aid in smear layer removal and improve surface cleanliness without producing excessive morphological damage. Several included studies, such as those by Saraswathi et al. [14], Viapiana et al. [15], and Lopes et al. [19], demonstrated that diode lasers produce consistent improvements in cleaning efficiency and facilitate the removal of organic residues and debris from the root canal walls. This is in agreement with earlier in-vitro research showing that diode lasers enhance the action of chelating agents and reduce the presence of residual smear layer in comparison to conventional irrigation alone [17,18,21,26]. The ability of diode lasers to penetrate deeper into dentinal tubules and activate irrigants through photothermal and photomechanical effects likely contributes to these observed benefits. Importantly, when diode lasers are applied in continuous or pulsed modes with moderate energy outputs, they appear to preserve the mineral integrity of dentin, as evidenced by stable calcium-to-phosphorus (Ca/P) ratios and minimal surface alterations [20,25]. These findings are consistent with the literature on the safe energy thresholds for diode lasers in endodontic use, which highlight the importance of maintaining power levels below the critical threshold that induces thermal damage [19,22].

Erbium lasers: Erbium-based lasers, specifically Er:YAG and Er,Cr:YSGG, showed notable promise for smear layer removal and dentin decontamination. These wavelengths (2,780 nm for Er,Cr:YSGG and 2,940 nm for Er:YAG) exhibit strong absorption in water, producing microexplosions that effectively dislodge debris from canal walls without substantial increases in temperature [6,9-11]. Studies such as those by Al-Omari and Palamara [16], Mobarak et al. [24], and de Menezes et al. [25] demonstrated that these systems, particularly in PIPS and SWEEPS modes, can achieve superior irrigant activation, improve penetration depth, and maintain or even enhance dentin microhardness. These findings align closely with recent meta-analyses emphasizing the superiority of erbium-based laser-assisted irrigation over conventional ultrasonic activation in achieving cleaner canal walls and more complete smear layer removal, especially in the apical third [10-12]. Moreover, the inclusion of fluoride or chelating solutions during laser activation, as explored by de Menezes et al. [25], revealed synergistic effects, with improved acid resistance and reduced hardness loss following demineralization cycles. This demonstrates the potential of laser-fluoride interaction as an adjunctive treatment for reinforcing dentin against caries and erosion [8,23].

Nd:YAG lasers: Nd:YAG lasers (1064 nm), despite their popularity in earlier decades, were associated with decreased microhardness and pronounced morphological changes in several studies, such as those by Al-Omari and Palamara [16] and de Macedo et al. [18]. The high penetration depth and strong photothermal effects of this wavelength may result in localized melting and recrystallization of dentin surfaces, leading to crater formation and structural weakening. This finding echoes earlier concerns in the literature that Nd:YAG irradiation, when used at high power densities, can cause undesirable thermal damage and compromise the mechanical integrity of dentin [2,18]. The data from this review therefore suggest that while Nd:YAG lasers remain effective for bacterial reduction and disinfection, their application for surface modification should be approached with caution and optimized for lower power and shorter exposure durations [16,18].

Optimization of Laser Parameters

In interpreting the broader implications of these findings, it becomes clear that the safety and efficacy of laser applications in endodontics depend on balancing the benefits of enhanced cleaning with the preservation of dentin integrity. The consistent improvement in smear layer removal across low- and moderate-bias studies indicates that laser-assisted irrigation can enhance traditional chemomechanical preparation methods [5,6,24]. Moreover, the preservation of dentin microhardness and fracture resistance, especially when diode and erbium lasers are used at appropriate settings, suggests that these techniques may improve the long-term durability of treated teeth [20,25]. These findings resonate with clinical goals emphasizing minimally invasive and biologically conservative endodontic strategies [3,5,7]. Furthermore, recent developments in laser pulse modulation, such as interval diode operation or photoacoustic streaming in Er:YAG systems, appear particularly promising for reducing microcrack formation and preventing thermal stress accumulation [20,24]. The ability to achieve superior cleaning with minimal structural compromise makes these newer laser protocols an attractive adjunct to conventional irrigation [11,12,25].

Limitations

Despite these encouraging outcomes, several limitations must be acknowledged regarding the evidence base. Most included studies were in vitro experiments conducted under controlled conditions that may not fully replicate the complexities of the clinical environment [19,23]. The use of extracted human or bovine teeth introduces variability in dentin composition and response to laser energy. Additionally, small sample sizes and inconsistent standardization of laser parameters limit the generalizability of the findings [6,9,10]. Only a minority of studies included blinded assessors or randomization, and very few reported confidence intervals or effect sizes, restricting quantitative comparison [4,22]. Furthermore, the lack of long-term aging or fatigue testing prevents assessment of the durability of laser-induced changes over time [23,25].

The review process itself has certain limitations. Although multiple databases were searched comprehensively, gray literature and unpublished studies were excluded, introducing the possibility of publication bias [4]. The heterogeneity in laser types, power settings, and outcome measures also precluded meta-analysis, necessitating reliance on narrative synthesis [5]. Finally, while risk of bias was carefully assessed, inter-reviewer agreement was not statistically quantified, which could influence the objectivity of some evaluations [4].

From a practical standpoint, these findings suggest that laser activation, especially using diode or erbium-based systems, should be considered as a safe and effective adjunct to conventional endodontic irrigation [5,6,7,11,25]. Clinicians should, however, adhere to established safety parameters and manufacturer recommendations to avoid overheating or overexposure [16,20]. From a policy perspective, dental education programs and clinical guidelines could benefit from incorporating standardized protocols for laser-assisted irrigation and dentin conditioning [3,5].

Implications for practice, policy, and future research

For future research, high-quality randomized controlled trials are essential to confirm these in vitro findings in vivo, with standardized reporting of laser parameters, energy settings, and temperature monitoring [4,6,9]. Long-term studies assessing microleakage, bond strength, and post-treatment structural resilience will be key to determining the clinical relevance of these techniques [9,10,12,25]. As laser technology continues to evolve, integrating controlled photoacoustic activation within endodontic protocols holds significant promise for advancing treatment efficacy while preserving dentin structure and promoting long-term tooth integrity [5,11,24,25].

## Conclusions

This systematic review aimed to evaluate the effects of various laser systems on dentin microhardness, smear layer removal, and surface characteristics. The findings indicate that diode and erbium-based lasers, when applied under controlled parameters, effectively enhance smear layer removal and preserve dentin integrity. Although Nd:YAG and high-powered Er, Cr:YSGG lasers may cause structural alterations, optimized laser protocols show promising potential as adjunctive tools for improving endodontic cleaning efficiency and dentin preservation.
